# Circulating suPAR in Relation to Renal and Cardiovascular Involvement in Systemic Lupus Erythematosus: A Pilot Study

**DOI:** 10.3390/ijms27209200

**Published:** 2026-10-16

**Authors:** Filomena Napolitano, Ilaria Mormile, Francesca Cevini, Amato de Paulis, Francesca Wanda Rossi, Nunzia Montuori

**Affiliations:** 1 Department of Translational Medical Sciences, University of Naples Federico II, Corso Umberto I 40, 80138 Naples, Italy; filomena.napolitano@unina.it (F.N.); ilaria.mormile@unina.it (I.M.); francesca.cevini@gmail.com (F.C.); depaulis@unina.it (A.d.P.);; 2 Center for Basic and Clinical Immunology Research (CISI), WAO Center of Excellence, University of Naples Federico II, 80131 Naples, Italy

**Keywords:** suPAR, lupus nephritis, cardiorenal marker, endothelial dysfunction, chronic inflammation

## Abstract

Soluble urokinase plasminogen activator receptor (suPAR) is an inflammatory biomarker associated with immune activation, endothelial dysfunction, and chronic inflammation. Increasing evidence suggests that suPAR may be involved in renal and cardiovascular injury and may reflect cumulative organ damage in systemic lupus erythematosus (SLE). Serum suPAR levels were measured in patients with SLE and healthy controls. Within the SLE cohort, suPAR levels were further evaluated according to the presence or absence of organ involvement. In this pilot study, serum suPAR levels were significantly higher in patients with SLE than in healthy controls. Within the SLE cohort, patients with renal involvement and those with reduced renal function had higher suPAR levels than their respective comparison groups. Similarly, patients with cardiovascular involvement showed significantly higher suPAR levels than SLE patients without cardiovascular involvement and healthy controls. However, serum suPAR levels were not significantly correlated with estimated glomerular filtration rate. Circulating suPAR levels are elevated in SLE and may be particularly associated with renal and cardiovascular involvement. These findings support the potential role of suPAR as a biomarker of cardiorenal involvement in SLE, although its relationship with renal function and clinical outcomes requires validation in larger prospective cohorts.

## 1. Introduction

Systemic lupus erythematosus (SLE) is a chronic autoimmune disorder characterized by diverse clinical manifestations, an unpredictable clinical course, and fluctuating periods of disease activity [1]. Its pathogenesis is complex and multifactorial, involving impaired clearance of apoptotic cells, dysregulation of innate and adaptive immune responses, complement activation, immune complex deposition, and subsequent tissue inflammation [2,3]. SLE can affect multiple organ systems, including the mucocutaneous, musculoskeletal, hematologic, pulmonary, cardiac, and renal systems [4,5,6,7]. Renal involvement is a major complication of SLE, with lupus nephritis (LN) occurring in nearly 40% of patients [8]. Among those affected, approximately 10% progress to end-stage kidney disease within 10 years [9]. The 2019 EULAR/ACR classification criteria are currently the most widely adopted framework for SLE classification, with excellent sensitivity and specificity [6]. However, the heterogeneity of SLE manifestations and fluctuating disease activity remain major challenges for diagnosis, prognostic stratification, and disease monitoring [10]. Conventional biomarkers, including anti-dsDNA antibodies and complement components (C3 and C4), are widely used to monitor disease activity, but their association with flares, prognosis, and specific organ involvement is inconsistent, highlighting the need for additional biomarkers to improve risk stratification and disease monitoring [11,12].

Recently, soluble urokinase plasminogen activator receptor (suPAR) has emerged as a promising biomarker in SLE, with potential applications in disease monitoring and prognostic stratification [13,14]. Beyond its role as a biomarker, suPAR has been implicated in several physiological and pathological processes, including inflammation, endothelial dysfunction, thrombosis, and tissue injury [15,16]. In patients with SLE, circulating suPAR levels have been reported to be higher than in healthy controls and to correlate with cumulative organ damage, although they do not appear to consistently reflect current disease activity [13]. Moreover, in the international SLICC Inception Cohort, baseline suPAR levels were 46.8% higher in patients who subsequently developed substantial organ damage (SDI ≥ 2) over 5 years, and suPAR independently predicted damage accrual (OR 1.14, 95% CI 1.03–1.26) [14].

The potential clinical relevance of suPAR may be particularly important in renal and cardiovascular disease. Increased circulating suPAR concentrations have been reported in patients with lupus nephritis and associated with proteinuria, with experimental data suggesting a potential role in podocyte dysfunction and altered integrin-dependent signaling [17]. More broadly, elevated suPAR levels have been associated with renal dysfunction, cardiovascular disease, and adverse cardiovascular outcomes, supporting a possible link between suPAR, endothelial injury, and organ damage [16,18]. However, data specifically addressing the relationship between circulating suPAR levels and renal and cardiovascular involvement in patients with SLE remain limited.

Herein, to further examine the roles of suPAR as a valuable biomarker in SLE, we evaluated serum suPAR levels in patients with SLE. Moreover, we analyzed their correlations with major clinical parameters.

## 2. Results

### 2.1. Baseline Characteristics and Laboratory Results of the Study Population

Sixteen adult patients [1 male (6.25%) and 15 females (93.75%)] diagnosed with SLE were enrolled in this study. The cohort was White-Caucasian. The average age at enrollment was 44.86 ± 13.41 years (22–70). The mean disease duration of SLE was 222 (156–333) months. Clinical features in our cohort of patients with SLE are summarized in Table 1.

Regarding disease involvement, from SLE diagnosis until last available follow-up, 7 (43.75%) patients had articular involvement (AI), 11 (68.75%) muco-cutaneous involvement (MCI), 7 (43.75%) hematological involvement (HI), 6 (37.5%) constitutional involvement (CI), 6 (37.5%) renal involvement (RI), 6 (37.5%) cardiovascular involvement (CVD), 3 (18.75%) neurological involvement (NI), and 1 (6.25%) gastrointestinal involvement (GI). At the time of the assessment, most patients exhibited well-controlled SLE. The median SLEDAI-2K score was 1 (0−2), consistent with low disease activity. Cardiovascular risk factors in the study cohort are reported in Table 1, including BMI, hypertension, dyslipidemia, diabetes mellitus, and smoking status. Notably, none of the patients had diabetes mellitus, only one patient was a smoker, and the other cardiovascular comorbidities were generally mild and under good clinical control at the time of enrollment.

The cohort was selected within the context of a pilot, exploratory study, with the aim of investigating circulating suPAR levels in patients with SLE characterized by low disease activity and thereby minimizing the potential influence of active systemic inflammation on suPAR levels. Importantly, this favorable disease status was achieved and maintained under active treatment, reflecting effective on-treatment disease control. Details of the therapeutic regimes are summarized in Table 2.

### 2.2. Serum Concentrations of suPAR in SLE Patients

**Figure 1:**
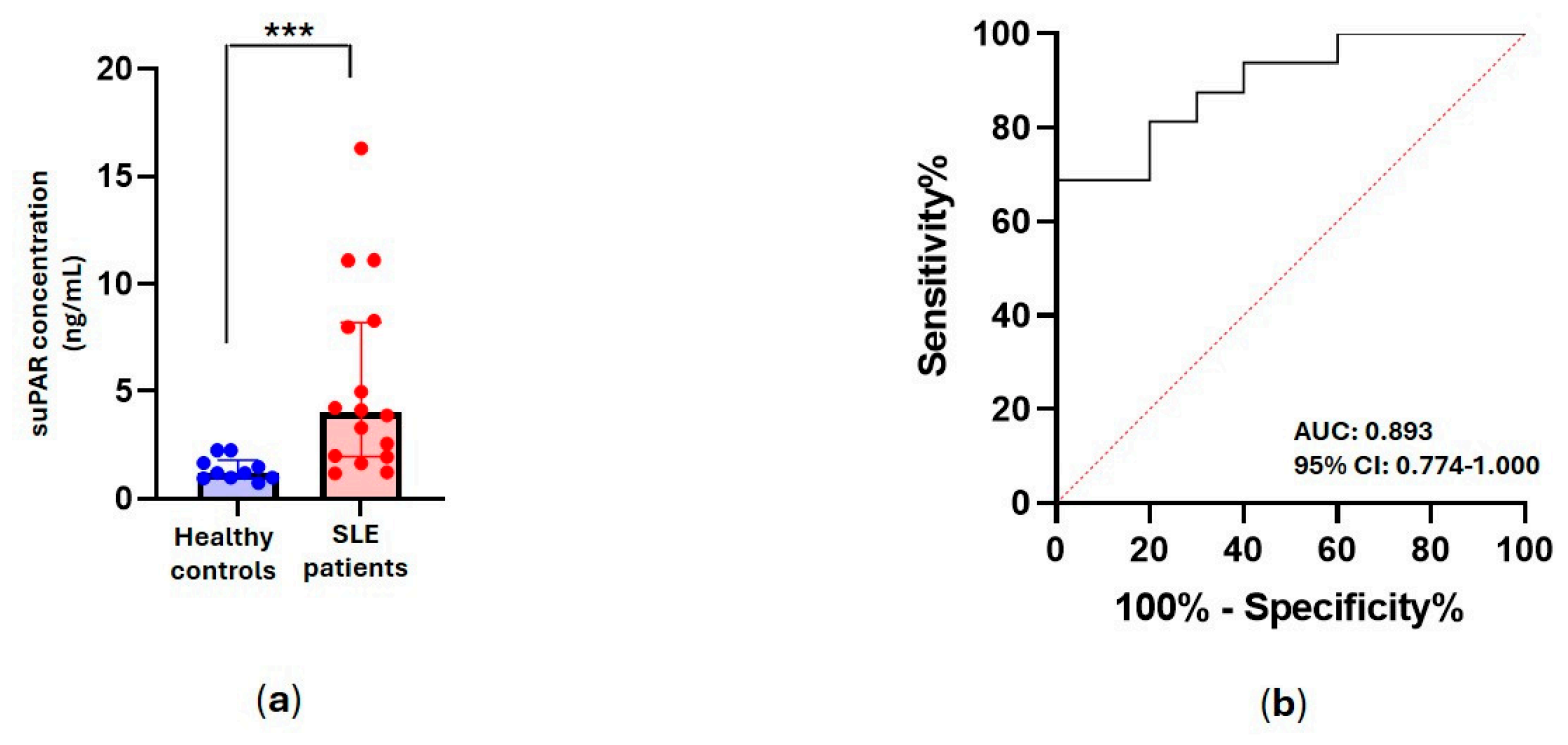
Analysis of serum concentrations of suPAR in SLE patients. (**a**) Serum suPAR concentrations in healthy controls (*n* = 10) and SLE patients (*n* = 16). Data are presented as median (IQR): 1.204 (0.9811.816) ng/mL in healthy controls and 3.998 (1.969-8.213) ng/mL in SLE patients. Statistical significance was assessed using the Mann–Whitney U test (*** *p* = 0.0004). (**b**) ROC curve analysis showing the diagnostic performance of serum suPAR for discriminating SLE patients from healthy controls.

Serum suPAR levels were measured in healthy controls (*n* = 10; 6 women and 4 men; mean age, 47.3 ± 14.8 years; range, 25–67 years) and patients with SLE (*n* = 16). Serum suPAR concentrations were compared between the two groups using the Mann–Whitney U test. As shown in Figure 1a, patients with SLE had significantly higher serum suPAR concentrations than healthy controls (5.366 ± 4.394 vs. 1.376 ± 0.538 ng/mL, respectively; median, 3.998 [IQR, 1.992–8.060] vs. 1.204 [IQR, 0.992–1.626] ng/mL; Mann–Whitney U = 17, *p* = 0.0004). The magnitude of the difference was large, as indicated by a rank-biserial correlation of 0.79. Serum suPAR concentrations in healthy controls were consistent with the reference range reported by the assay manufacturer, supporting the reliability of the measurements obtained in this study.

In this small exploratory cohort, ROC curve analysis showed an apparent ability of serum suPAR levels to discriminate patients with SLE from healthy controls, with an AUC of 0.893 (95% CI, 0.774–1.000; *p* = 0.0009; Figure 1b). A data-derived cutoff of >1.813 ng/mL yielded the highest Youden’s index (0.613), with a sensitivity of 81.25% and a specificity of 80.00%. Given the limited sample size, these estimates and the proposed cutoff should be considered exploratory and hypothesis-generating only and should not be interpreted as evidence of diagnostic performance or clinical utility. Independent validation in larger cohorts is required.

### 2.3. Association of Serum suPAR with Renal and Cardiovascular Involvement in SLE Patients

**Figure 2:**
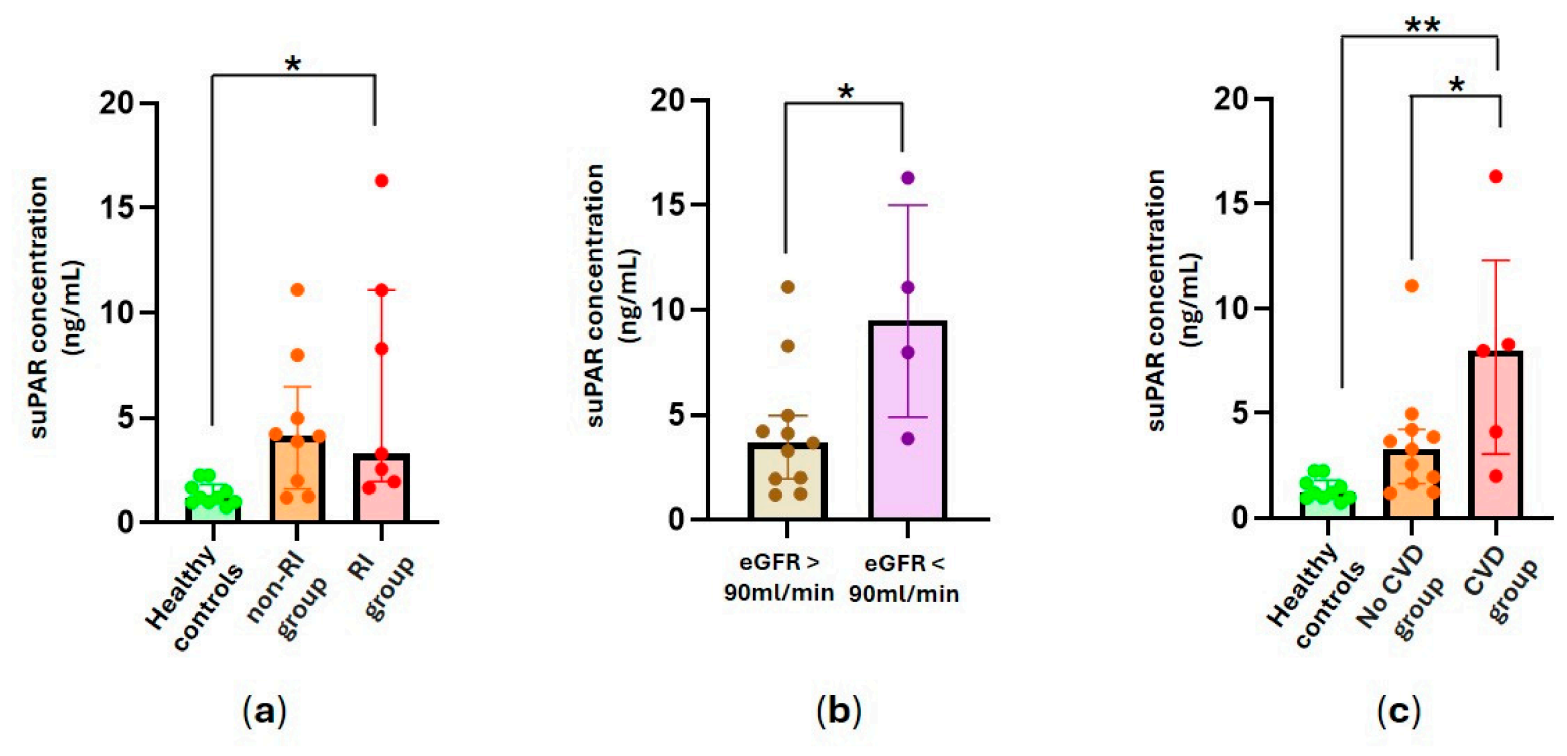
The association of serum suPAR levels with renal and cardiovascular involvement. (**a**) Serum suPAR concentration in healthy controls (*n* = 10), SLE patients without renal involvement (non-RI group; *n* = 9), and SLE patients with renal involvement (RI group; *n* = 7). (**b**) Serum suPAR levels in patients with SLE stratified according to estimated glomerular filtration rate (eGFR) categories (<90 mL/min/1.73 m^2^, *n* = 12 vs. ≥90 mL/min/1.73 m^2^, *n* = 4). (**c**) Serum suPAR concentration in healthy controls (*n* = 10), SLE patients without cardiovascular manifestations (non-CVD; *n* = 11), and SLE patients with cardiovascular manifestations (CVD group; *n* = 5). * *p* ≤ 0.05; ** *p* ≤ 0.001.

Serum suPAR levels were compared among healthy controls, SLE patients without RI (non-RI), and SLE patients with RI using one-way ANOVA followed by Tukey’s multiple-comparisons test (Figure 2a). The overall analysis revealed a statistically significant difference among the three groups (F(2, 23) = 4.662, *p* = 0.020, η^2^ = 0.289). Post hoc analysis showed that serum suPAR concentrations were higher in patients with RI than in healthy controls (6.450 ± 5.635 ng/mL vs. 1.376 ± 0.538 ng/mL, respectively; mean difference, 5.074 ng/mL; 95% CI, 0.7855–9.362; adjusted *p* = 0.0184). The difference between healthy controls and SLE patients without RI was not statistically significant (4.523 ± 3.251 vs. 1.376 ± 0.538 ng/mL, respectively; mean difference, 3.147 ng/mL; 95% CI, −0.8511 to 7.146; adjusted *p* = 0.1420). Similarly, the difference between SLE patients without RI and those with RI was not statistically significant (4.523 ± 3.251 vs. 6.450 ± 5.635 ng/mL, respectively; mean difference, 1.927 ng/mL; 95% CI, −2.459 to 6.312; adjusted *p* = 0.5236).

We further investigated the association between serum suPAR levels and renal function. SLE patients with reduced renal function (eGFR < 90 mL/min/1.73 m^2^; *n* = 4) had higher serum suPAR concentrations than those with preserved renal function (eGFR ≥ 90 mL/min/1.73 m^2^; *n* = 11) (9.817 ± 5.243 vs. 4.189 ± 3.064 ng/mL, respectively; mean difference, 5.628 ng/mL; 95% CI, 0.9814–10.27; Cohen’s d = 1.53; *p* = 0.0213; unpaired t-test). However, Spearman correlation analysis did not demonstrate a significant association between serum suPAR levels and eGFR (rho = −0.042, *p* = 0.444.

We next assessed the association between serum suPAR levels and CVD using one-way ANOVA followed by Tukey’s multiple-comparisons test (Figure 2c). For this analysis, CVD was defined according to documented cardiovascular events/manifestations, while cardiovascular risk factors alone were not considered sufficient to classify patients as having CVD. The overall analysis revealed a statistically significant difference among the three groups (F(2, 23) = 7.768, *p* = 0.0026, η^2^ = 0.403). Post hoc analysis showed that serum suPAR concentrations were higher in SLE patients with CVD than in healthy controls (7.741 ± 5.745 vs. 1.376 ± 0.538 ng/mL, respectively; mean difference, 6.365 ng/mL; 95% CI, 2.320–10.41; Tukey-adjusted *p* = 0.0018). Serum suPAR levels were also higher in patients with CVD than in SLE patients without CVD (7.741 ± 5.745 vs. 3.611 ± 2.784 ng/mL, respectively; mean difference, 4.130 ng/mL; 95% CI, 0.1464–8.113; adjusted *p* = 0.0412). In contrast, the difference between healthy controls and SLE patients without CVD was not statistically significant (1.376 ± 0.538 vs. 3.611 ± 2.784 ng/mL, respectively; mean difference, 2.235 ng/mL; 95% CI, −0.9920 to 5.462; adjusted *p* = 0.2142). Given the small sample size and exploratory nature of the analysis, these findings should be interpreted cautiously.

### 2.4. Association of Serum suPAR with Other Clinical Manifestations in SLE Patients

Associations between serum suPAR levels and other clinical manifestations, including CI, AI, and NI, were evaluated in SLE patients. For each clinical manifestation, serum suPAR levels were compared among healthy controls and SLE patients with or without the corresponding manifestation using one-way ANOVA followed by Tukey’s multiple-comparisons test.

**Figure 3:**
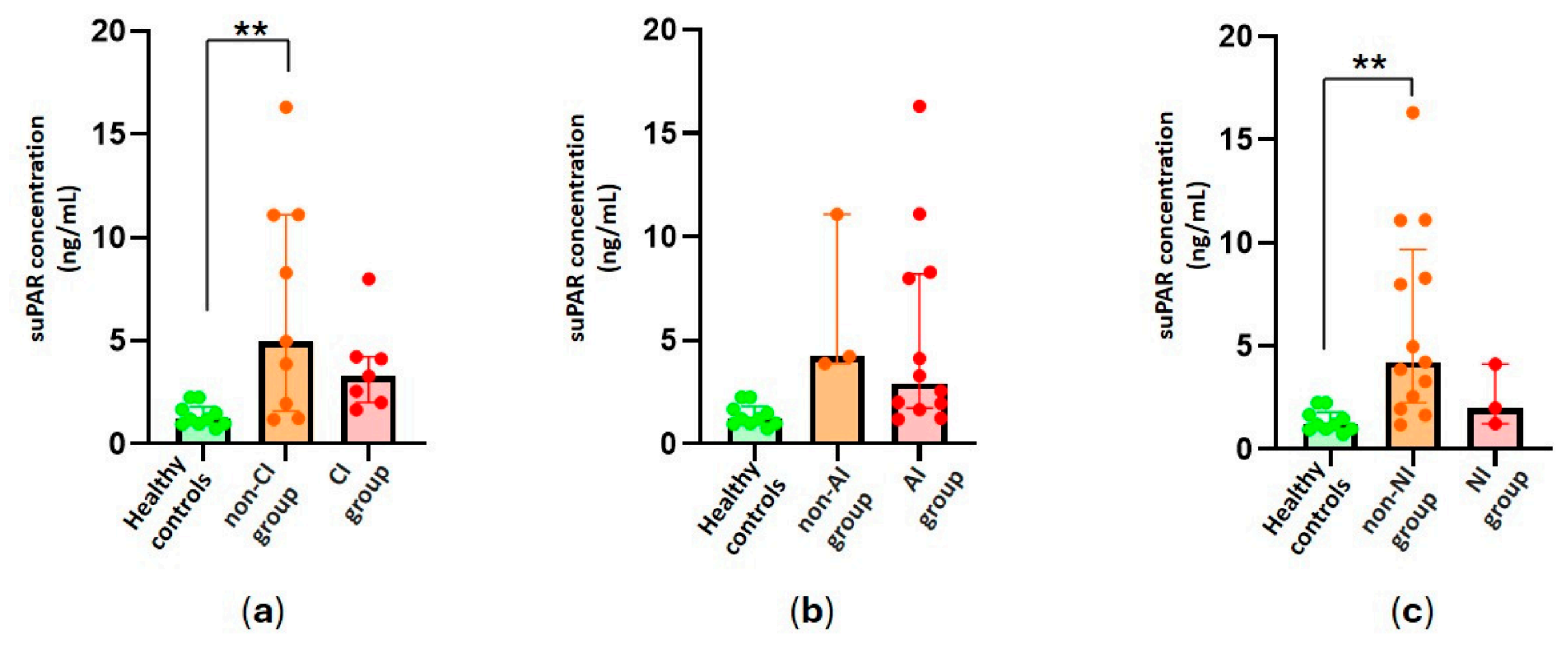
Analysis of serum concentrations of suPAR in SLE patients stratified according to multi-organ involvement. (**a**) Serum suPAR concentrations in healthy controls (*n* = 10), SLE patients without constitutional involvement (non-CI group; *n* = 9), and SLE patients with constitutional involvement (CI group; *n* = 7). (**b**) Serum suPAR levels in healthy controls (*n* = 10), SLE patients without articular involvement (non-AI group; *n* = 3), and SLE patients with articular involvement (AI group; *n* = 12). (**c**) Serum suPAR concentration in healthy controls (*n* = 10), SLE patients without neurological involvement (non-NI group; *n* = 13), and SLE patients with neurological involvement (NI group; *n* = 3). ** *p* ≤ 0.001.

As shown in Figure 3a, the overall analysis revealed a statistically significant difference among the three groups (F(2, 23) = 5.942, *p* = 0.0083, η^2^ = 0.341). Serum suPAR levels were significantly higher in SLE patients without CI than in healthy controls (6.671 ± 5.331 vs. 1.376 ± 0.538 ng/mL; mean difference, 5.294 ng/mL; 95% CI, 1.446–9.143; adjusted *p* = 0.006). In contrast, no significant difference was observed between SLE patients with CI and healthy controls (3.689 ± 2.134 vs. 1.376 ± 0.538 ng/mL; mean difference, 2.313 ng/mL; 95% CI, −1.815 to 6.441; adjusted *p* = 0.356). Likewise, serum suPAR levels did not differ significantly between SLE patients without CI and those with CI (mean difference, 2.981 ng/mL; 95% CI, −1.240 to 7.203; adjusted *p* = 0.202).

For AI (Figure 3b), the overall analysis revealed a statistically significant difference among the three groups (F(2, 22) = 3.836, *p* = 0.0372, η^2^ = 0.259). However, no statistically significant differences were observed in the Tukey-adjusted pairwise comparisons. The mean difference between healthy controls and SLE patients without AI was 5.020 ng/mL (95% CI, 0.9675–11.01; adjusted *p* = 0.112), whereas the mean difference between healthy controls and SLE patients with AI was 3.766 ng/mL (95% CI, −0.1284 to 7.660; adjusted *p* = 0.059). No significant difference was observed between SLE patients without AI and those with AI (mean difference, 1.254 ng/mL; 95% CI, −4.617 to 7.125; adjusted *p* = 0.854).

Finally, for NI, the overall analysis revealed a statistically significant difference among the three groups (F(2, 23) = 5.697, *p* = 0.0098, η^2^ = 0.331). Serum suPAR levels were significantly higher in SLE patients without NI than in healthy controls (6.038 ± 4.600 vs. 1.376 ± 0.538 ng/mL; mean difference, 4.662 ng/mL; 95% CI, 1.114–8.211; adjusted *p* = 0.0087). However, no significant difference was observed between SLE patients with NI and healthy controls (2.454 ± 1.490 vs. 1.376 ± 0.538 ng/mL; mean difference, 1.078 ng/mL; 95% CI, −4.476 to 6.631; adjusted *p* = 0.879). Similarly, the difference between SLE patients without NI and those with NI was not statistically significant (mean difference, 3.585 ng/mL; 95% CI, −1.819 to 8.988; adjusted *p* = 0.241).

Overall, these data showed significantly higher serum suPAR levels in SLE patients without CI and without NI compared with healthy controls, whereas no statistically significant differences were observed between SLE patients with and without CI, AI, or NI. In addition, the findings from the analyses of other clinical manifestations suggest that serum suPAR levels may be particularly associated with RI and CVD. Given the small sample sizes of the clinical subgroups, particularly those with AI and NI, these findings should be considered exploratory and interpreted with caution.

## 3. Discussion

In this study, we found that serum suPAR levels were higher in patients with SLE than in healthy controls, in line with previous observations [13,14]. ROC curve analysis provided preliminary evidence of an association between serum suPAR levels and SLE status in this small exploratory cohort. However, given the limited sample size, the cross-sectional design, and the absence of external validation, these findings should be considered hypothesis-generating only and should not be interpreted as evidence of diagnostic performance or clinical utility. Larger, independent and adequately powered studies are needed to validate these preliminary findings and to clarify the potential role of suPAR as a circulating biomarker in SLE.

A major finding of our study was the observation of higher serum suPAR levels in patients with RI. Patients with RI had higher suPAR concentrations than healthy controls, and, importantly, SLE patients with reduced renal function (eGFR < 90 mL/min/1.73 m^2^) showed significantly higher suPAR levels than those with preserved renal function. These findings are consistent with previous reports linking elevated suPAR levels to lupus nephritis and proteinuria and with experimental evidence suggesting that suPAR may contribute to podocyte dysfunction through altered integrin-dependent signaling [17]. Nevertheless, in our cohort, serum suPAR levels did not significantly correlate with eGFR as a continuous variable. Thus, our findings indicate an exploratory association between elevated suPAR levels and reduced renal function, but do not establish a linear relationship between circulating suPAR concentrations and eGFR. The discrepancy may reflect the small sample size and/or the heterogeneity of RI.

We also observed significantly higher serum suPAR concentrations in SLE patients with CVD compared with both SLE patients without CVD and healthy controls. Since CVD represents a major cause of morbidity and mortality in SLE [19,20], the identification of biomarkers associated with vascular involvement is of considerable clinical interest [21]. In addition, this finding is of particular interest given the established involvement of suPAR in inflammatory and vascular processes, including endothelial dysfunction, thrombosis, and tissue injury [16,22,23,24]. Previous studies in non-SLE populations have associated elevated suPAR levels with CVD and adverse cardiovascular outcomes, supporting the hypothesis that suPAR may reflect systemic vascular injury or an inflammatory milieu associated with cardiovascular complications [25,26]. Our findings are consistent with an association between higher suPAR levels and CVD in this cohort; however, they do not establish that suPAR specifically reflects cardiovascular involvement. However, the cross-sectional nature of our study does not allow us to determine whether elevated suPAR precedes or results from CVD.

A further consideration is the potential influence of clinical and biological factors that may independently affect circulating suPAR levels. In particular, renal function and systemic inflammation may represent important sources of confounding when interpreting the higher suPAR concentrations observed in patients with RI or CVD. Although our cohort was characterized by generally low disease activity, and we considered available clinical and laboratory parameters including eGFR, ESR, CRP, disease duration, and treatment exposure, the small sample size and the limited number of patients within the clinical subgroups did not allow for a reliable multivariable analysis. Therefore, the associations observed between suPAR levels and RI or CVD should be regarded as exploratory, unadjusted associations rather than evidence of organ-specific biomarker performance. In particular, the higher suPAR levels observed in patients with RI may partly reflect the known relationship between circulating suPAR and renal function, while the association with CVD may similarly be influenced by systemic inflammatory and vascular processes. Larger studies with adequate sample sizes and multivariable adjustment are needed to determine whether these associations remain independent of renal function, inflammation, and other potential confounders.

Interestingly, serum suPAR levels were significantly higher in SLE patients without CI and without NI compared with healthy controls, whereas no statistically significant differences were observed between SLE patients with and without CI, AI, or NI. These findings suggest that the increased circulating suPAR observed in SLE cannot be directly attributed to the presence of these individual clinical manifestations. Rather, suPAR levels may reflect broader systemic processes associated with SLE, including inflammatory, endothelial, or tissue-related alterations. The absence of consistent differences according to CI, AI, or NI is also compatible with previous evidence suggesting that suPAR may be more closely related to cumulative organ damage than to current disease activity [13,14]. In this context, the stronger associations observed with RI and CVD in our cohort should not be interpreted as evidence that suPAR is specifically responsive to renal or vascular injury, particularly given the potential confounding effects discussed above. However, given the small sample sizes of the clinical subgroups, particularly for AI and NI, these findings should be considered exploratory and interpreted with caution.

In conclusion, our findings confirm that circulating suPAR levels are elevated in SLE and identify exploratory associations with renal and cardiovascular involvement. However, given the small sample size, the cross-sectional design, and the potential influence of renal function, systemic inflammation, and other clinical factors, these associations should not be interpreted as evidence of organ-specific biomarker performance. The lack of a significant correlation with eGFR and the absence of consistent associations with other clinical manifestations further indicate that suPAR should not be regarded as a direct marker of disease activity or renal function. Rather, suPAR may reflect broader systemic inflammatory, tissue, or vascular processes in SLE. Larger, prospective and adequately powered studies with appropriate adjustment for potential confounders are warranted to determine the clinical relevance and independence of these preliminary associations.

## 4. Materials and Methods

### 4.1. Clinical and Laboratory Assessments

In this cross-sectional single-center cohort study, we evaluated 16 patients with SLE treated at the Division of Internal Medicine and Clinical Immunology of the University of Naples Federico II, Naples, Italy.

Inclusion criteria were an age ≥ 18 years, confirmed SLE diagnosis according to the SLICC 2012 [27] and/or ACR/EULAR 2019 classification criteria [6] and signature of the written informed consent. Exclusion criteria were pregnancy, breastfeeding, and other systemic autoimmune rheumatic diseases.

We enrolled 10 healthy Caucasian age- and gender-matched subjects as controls with no history of autoimmune or inflammatory diseases. Healthy control participants were recruited voluntarily.

Demographic and clinical data were retrieved from patient medical charts and diaries. We collected data regarding date of SLE onset and diagnosis, diagnostic delay, type of disease involvement considering all documented organ manifestations occurring at any time during the entire disease course (lifetime disease involvement), overall disease severity, disease activity at last follow-up, overall disease course, and SLE medication therapy details. Disease severity was defined according to the EULAR recommendations [6] as major organ threatening disease considering the worst disease manifestations occurring anytime during the whole medical history (nephritis, cerebritis, myelitis, pneumonitis, mesenteric vasculitis); and thrombocytopenia with ≥1 BILAG A manifestations. Disease activity at last available follow-up was assessed based on the Systemic Lupus Erythematosus Disease Activity Index 2000 (SLEDAI-2 K) score [28]. All SLE-specific treatments administered over the patient’s lifetime were considered in the evaluation of therapeutic history across the disease course, with a particular focus on glucocorticoids (GCs), hydroxycholoquine (HCQ), traditional disease-modifying antirheumatic drugs (tDMARDs), and biologic DMARDs (bDMARDs).

Laboratory measurements encompassed C-reactive protein (mg/dL), erythrocyte sedimentation rate value (mm/h), C3 (mg/dL), C4 (mg/dL), eGFR (mL/min/1.73 mq), 24 h proteinuria (mg/24 h), serum creatinine (mg/dL), fibrinogen (mg/dL), activated partial thromboplastin time (aPTT, ratio), prothrombin time expressed as international normalized ratio (PT-INR), blood urea nitrogen (BUN, mg/dL), gamma fraction of serum protein electrophoresis (%, g/dL), serum creatinine (mg/dL), and 24 h urine protein test (mg/day). The autoantibody profile was assessed at the Clinical Pathology Laboratory of the University of Naples Federico II, Naples, Italy. ANA were determined by indirect immunofluorescence (IIF), whereas anti-dsDNA and extractable nuclear antigen (ENA) antibodies were assessed by chemiluminescence immunoassay (CLIA). The ENA profile included anti-Ro/SSA, anti-La/SSB, anti-Sm, anti-RNP, anti-Scl-70, and anti-Jo-1 antibodies. Anti-centromere antibodies were also assessed by CLIA, whereas anti-histone antibodies were determined by ELISA. According to the laboratory reference ranges, anti-Ro/SSA, anti-La/SSB, anti-Sm, anti-RNP, anti-Scl-70, anti-Jo-1, and anti-centromere antibody levels < 10 UA/mL were considered negative. For anti-histone antibodies, levels < 20 UA/mL were considered negative, 20–25 UA/mL borderline, and >25 UA/mL positive. Anti-dsDNA positivity was defined according to the laboratory-specific reference range for the assay. The antiphospholipid antibody (aPL) profile included anticardiolipin (aCL) IgG and IgM antibodies, anti-β2 glycoprotein-I (β2GPI) IgG and IgM antibodies, and lupus anticoagulant (LAC). aCL IgG and IgM antibodies and β2GPI IgG and IgM antibodies were assessed by CLIA, and antibody levels < 20 U/mL were considered negative. LAC was evaluated by clotting assay, and a ratio < 1.20 was considered negative.

The study was conducted in accordance with the Declaration of Helsinki and approved by the Ethics Committee of the University of Naples Federico II (prot. no. 14/22, approved on 16 November 2022). Prior to study participation, patients signed an informed consent form.

### 4.2. Sample Collection

Fasting venous blood samples were collected from both patients and healthy controls. Blood was drawn into serum separator tubes and allowed to clot at room temperature for 2 h. The samples were then centrifuged at 1500× *g* for 20 min. The resulting serum was either analyzed immediately or divided into multiple aliquots and stored at −80 °C until further analysis, to avoid repeated freeze–thaw cycles.

### 4.3. Determination of suPAR

Serum suPAR concentrations were measured at baseline in all patients and controls using a clinically validated enzyme-linked immunosorbent assay (ELISA) kit (suPARnostic^®^ AUTO Flex ELISA; ViroGates, Birkerød, Denmark), according to the manufacturer’s instructions. Laboratory personnel performing the ELISA measurements were blinded to the participants’ clinical status. Briefly, the suPARnostic^®^ AUTO Flex ELISA is a double-monoclonal-antibody sandwich assay in which serum samples and peroxidase-conjugated anti-suPAR antibodies are mixed in a mixing plate before incubation in anti-suPAR-coated, optically clear microwells. The assay uses monoclonal mouse and rat antibodies directed against human suPAR DI–III. A volume of 15 μL of each serum sample was analyzed in a single well. Following incubation, tetramethylbenzidine (TMB) substrate solution was added, and the enzymatic reaction was terminated with 2 N sulfuric acid. Absorbance was measured at 450 nm within 30 min after stopping the reaction using a BGMR-1000 Microplate Reader (Hangzhou Bio-gener Technology Co., Ltd., Hangzhou, China). Serum sample concentrations were calculated according to the standard curve included in each plate. The intra-assay variation of the kit is given as 2.8% and the inter-assay variation as 9.2% [29].

### 4.4. Statistical Analysis

Statistical analyses were performed using GraphPad Prism 9 (GraphPad Software, La Jolla, CA, USA). Continuous variables were expressed as mean ± standard deviation (SD) or median and range, as appropriate, whereas categorical variables were presented as number (*n*) and percentage (%). The normality of data distribution was assessed using the Shapiro–Wilk test.

For comparisons between two independent groups, an unpaired Student’s *t*-test was used for normally distributed data, whereas the Mann–Whitney U test was used for non-normally distributed data. Comparisons involving three independent groups were performed using one-way analysis of variance (ANOVA), followed by Tukey’s multiple-comparisons test for pairwise comparisons. Adjusted *p* values and 95% confidence intervals (CIs) for pairwise differences were reported for multiple comparisons.

Correlations between continuous variables were assessed using Spearman’s rank correlation coefficient (r). Receiver operating characteristic (ROC) curve analysis was performed to evaluate the diagnostic performance of the parameters studied.

Given the small sample sizes and the multiple clinical subgroup analyses, subgroup analyses were considered exploratory, and their results were interpreted with caution. A two-sided *p* value ≤ 0.05 was considered statistically significant, with multiplicity-adjusted *p* values used for post hoc pairwise comparisons. In addition to statistical significance, effect sizes were calculated to quantify the magnitude of the observed effects. Eta squared (η^2^) was used for ANOVA, Cohen’s *d* for independent-samples *t*-tests, and rank-biserial correlation for Mann–Whitney U tests.

## Figures and Tables

**Table 1:** Clinical, laboratory, and treatment characteristics of the SLE cohort (N = 16). Data are presented as *n* (%), mean ± SD, or median (Q1–Q3), as appropriate. Current treatment regimens are reported at the last follow-up visit. SLEDAI-2K, Systemic Lupus Erythematosus Disease Activity Index 2000; ESR, erythrocyte sedimentation rate; eGFR, estimated glomerular filtration rate; CRP, C-reactive Protein; SPEP, serum protein electrophoresis. Cardiovascular involvement was defined as the presence of at least one documented cardiovascular manifestation or event occurring at any time during the disease course. Specific manifestations are reported as *n* (%) of the overall study population (N = 16); individual patients could present with more than one cardiovascular manifestation.

Patients’ Features	Overall (*n* = 16)
Female gender (*n*, %)	15 (93.7)
Caucasian ethnicity (*n*, %)	16 (100)
Age, years (mean ± SD, range)	44.8 ± 13.4 (22–70)
Body mass index (kg/m^2^, mean ± SD, range)	25.8 (23.39–32.77)
Hypertension (*n*, %)	6 (37.5)
Dyslipidemia (*n*, %)	5 (31.25)
Diabetes (*n*, %)	0 (0%)
Smokers (*n*, %)	1 (6.25)
Non-Smokers (*n*, %)	14 (87.5)
Former smokers (*n*, %)	1 (6.25)
Disease duration (months; median (Q1–Q3))	222 (156–333)
Diagnostic delay (months; median (Q1–Q3))	24 (0–60)
SLEDAI 2k (median (Q1–Q3))	1 (0–2)
ESR (mm/h; mean ± SD, range)	11.8 ± 7.9 (2–25)
CRP (mg/dL; median (Q1–Q3))	0.1 (0–0.7)
eGFR (mL/min/1.73 m^2^; mean ± SD, range)	94.3 ± 21 (49.2–114)
Serum creatinine (mg/dL; mean ± SD, range)	0.8 ± 0.17 (0.64–1.26)
24 h proteinuria (mg/24 h, median (Q1–Q3))	150 (100–440)
SPEP, gamma fraction (g/dL; median (Q1–Q3))	9.52 (1.27–12)
SPEP, gamma fraction (%; median (Q1–Q3))	14.1 (12.8–17.1)
C3 (g/L; median (Q1–Q3))	0.9 (0–0.9)
C4 (g/L; median (Q1–Q3))	0.1 (0.1–0.2)
Any antiphospholipid antibody positivity, *n* (%)	6 (37.5)
Lupus anticoagulant (ratio, median (Q1–Q3))	0.86 (0.78–0.99)
IgM anticardiolipin antibodies (U/mL, median (Q1–Q3))	1.9 (0.4–10.7)
IgG anticardiolipin antibodies (U/mL, median (Q1–Q3))	5.40 (1.8–13.6)
IgM anti-β2 glycoprotein-I antibodies (U/mL, median (Q1–Q3))	1 (0.1–1.6)
IgG anti-β2 glycoprotein-I antibodies (U/mL, median (Q1–Q3))	6.90 (2.8–18.9)
Any cardiovascular manifestation	6 (37.5%)
Left ventricular diastolic dysfunction	2 (12.5)
Left atrial dilatation	1 (6.25)
Valvular disease	6 (37.5%)
Pericarditis	6 (37.5%)
Chronic ischemic cerebral changes	1 (6.25)

**Table 2:** Treatment characteristics of the SLE cohort (N = 16). ANI: anifrolumab; AZA: azathioprine; BLM: belimumab; HCQ: hydroxychloroquine; MMF: mycophenolate mofetil; MTX: methotrexate; PND: prednisone; VCS: voclosporin.

**Treatment at the last follow-up visit**	
HCQ monotherapy (*n*, %)	4 (25)
MMF monotherapy (*n*, %)	1 (6.25)
HCQ + PDN (<20 mg/day) (*n*, %)	1 (6.25)
HCQ + MTX (*n*, %)	1 (6.25)
HCQ + MMF (*n*, %)	2 (12.5)
HCQ + BLM (*n*, %)	2 (12.5)
HCQ +ANI (*n*, %)	1 (6.25)
PDN (<20 mg/day) + MMF (*n*, %)	1 (6.25)
HCQ + PDN (<20 mg/day) + BLM (*n*, %)	2 (12.5)
PDN (<20 mg/day) + MMF+ BLM (*n*, %)	1 (6.25)
**Lifetime treatment exposure**	
HCQ + PDN (*n*, %)	4 (25)
HCQ + MTX + PDN (*n*, %)	1 (6.25)
HCQ + MMF + PDN (*n*, %)	2 (12.5)
HCQ + AZA + PDN (*n*, %)	1 (6.25)
HCQ + BLM + PDN (*n*, %)	2 (12.5)
HCQ + MMF + BLM + PDN (*n*, %)	3 (18.75)
HCQ + BLM + ANI + PDN (*n*, %)	1 (6.25)
MMF + VCS + PDN (*n*, %)	1 (6.25)
HCQ + MMF + BLM + VCS + PDN (*n*, %)	1 (6.25)

## Data Availability

The data underlying this article cannot be shared publicly due to the privacy of individuals that participated in the study. The data will be shared on reasonable request to the corresponding author.
